# Supplementary material for: Chromothripsis during telomere crisis is independent of NHEJ, and consistent with a replicative origin
Source: Genome Res. 2019 May;29(5):737–49. doi: 10.1101/gr.240705.118 (PMC6499312; doi:10.1101/gr.240705.118)
Supplement: Supplemental Material [file supp_gr.240705.118_Supplemental_file_1.zip › contigs/annotated_contigs/DB57/contig.2.DB57_length_808_mean_cov_9.62128712871.docx]

**DB57_length_808_mean_cov_9.62128712871**

```
TGTAAGTGTTGTGCCTTCTGTTTGTCACAGTAATCCTATGAGGAAGGTATTATTCTTTCCATCTTCAAAGGAGGAAAGTGAGGCTTAGA
 >chr7:147238144-147238497 - E=2e-197 p=1e-02
GCAATTAAAGTAACAGAAGGCTATGGAGCTTGAAAGTAATGGAATAGAATGAGATTTGAGCCTATAGCCCCTGTTCCTAGCCACTGCAT

TATACTAAGAGTCAAAGATATCATATGGCTTACTCACTCAAAGTCACTCACTAATTTAGTGTCAATTACCAGACCAGAATTTGGGTGTT

CTAACTGTTACCATCTTCCCCCTATATCGTATACATTTTTAAAAGAAGTAGATGTTAAACATAAATGGAGTTTTCCTTGCTATAAG|TT

TAACCTAACACTCAATTAAGTGTATGAAAAGTGCTGAGATTTATCCATTAATTTTTCTCATAAGAAACTAGATCACGTATTTCTTTTAC
>chr7:146137405-146137838 + E=1e-230
CAAAATTCTATTTGACATTCAACTTAAAATCCAAAGAGTGTAATGAAAGCCCTTAAGATAATGACTGTGAAATAGCAATATAGCCTGAC

GTTTAATGAAGTGGATGGGTATGTCTCTTACCTAAAGAGAAGATGTGAAAATTTAAAGTCACACTGGTTATGCTGTCTGTGTGTATATC

TTCATTTTTAAAAAAATCAGACTCTTGAGAATTTCATTACCTAGCCCTCCACCAAAACAATGCATCTGCGTAAATATTTCACAATTTTC

CATTGTTTTAAAGTTGGCTGATGAGAGGTTACTAAGTACTGTGTGGAATTATTCCAAAATCTTTTATACTACAAC|AAAGAAGTAAAAA

GGGCTCTAG
```
